# Iron overload promotes mitochondrial fragmentation in mesenchymal stromal cells from myelodysplastic syndrome patients through activation of the AMPK/MFF/Drp1 pathway

**DOI:** 10.1038/s41419-018-0552-7

**Published:** 2018-05-03

**Authors:** Qingqing Zheng, Youshan Zhao, Juan Guo, Sida Zhao, Chengming Fei, Chao Xiao, Dong Wu, Lingyun Wu, Xiao Li, Chunkang Chang

**Affiliations:** Department of Hematology, Shanghai Jiao Tong University Affiliated Sixth People’s Hospital, Shanghai, 200233 China

## Abstract

Iron overload (IO) has been reported to contribute to mesenchymal stromal cell (MSC) damage, but the precise mechanism has yet to be clearly elucidated. In this study, we found that IO increased cell apoptosis and lowered cell viability in MSCs, accompanied by extensive mitochondrial fragmentation and autophagy enhancement. All these effects were reactive oxygen species (ROS) dependent. In MSCs with IO, the ATP concentrations were significantly reduced due to high ROS levels and low electron respiratory chain complex (ETC) II/III activity. Reduced ATP phosphorylated AMP-activated protein kinase (AMPK). Activation of AMPK kinase complexes triggered mitochondrial fission. Moreover, gene knockout of AMPK via CRISPR/Cas9 reduced cell apoptosis, enhanced cell viability and attenuated mitochondrial fragmentation and autophagy caused by IO in MSCs. Further, AMPK-induced mitochondrial fragmentation of MSCs with IO was mediated via phosphorylation of mitochondrial fission factor (MFF), a mitochondrial outer-membrane receptor for the GTPase dynamin-related protein 1 (Drp1). Gene knockdown of MFF reversed AMPK-induced mitochondrial fragmentation in MSCs with IO. In addition, MSCs from IO patients with myelodysplastic syndrome (MDS) showed increased cell apoptosis, decreased cell viability, higher ROS levels, lower ATP concentrations and increased mitochondrial fragmentation compared with MSCs from non-IO patients. In addition, iron chelation or antioxidant weakened the activity of the AMPK/MFF/Drp1 pathway in MDS-MSCs with IO from several patients, accompanied by attenuation of mitochondrial fragmentation and autophagy. Taken together, the AMPK/MFF/Drp1 pathway has an important role in the damage to MDS-MSCs caused by IO.

## Introduction

Myelodysplastic syndrome (MDS) is a heterogeneous group of clonal disorders derived from haematopoietic stem and progenitor cells, and is characterized by ineffective bone marrow (BM) haematopoiesis, peripheral blood cytopaenias and a risk of progression to acute myeloid leukaemia^1^. Iron overload (IO) occurs in 50–80% of MDS patients and can cause damage of haematopoietic stem/progenitor cells (HSPCs), having an impact on both the overall and leukaemia-free survival in MDS patients^2–6^. Recently, accumulating evidence has revealed that MDS-derived mesenchymal stromal cells (MDS-MSCs) with IO were deficient in proliferation and differentiation, and exhibited increased apoptosis and senescence, which may also contribute to MDS progression^6–8^.

MSCs are key functional components of the BM microenvironment and have an important role in supporting and regulating HSPCs via secretion of haematopoietic cytokines, chemokines and adhesion factors^8,9^. In addition to their supportive effects, MSCs may also facilitate damage of HSPCs under some pathological circumstances. Some research has revealed that under the IO conditions, the haematopoiesis-supporting capacity of MSCs is weakened and the osteogenic/adipogenic differentiation potential is decreased^8,10^. As reported, high levels of reactive oxygen species (ROS) caused by IO may be a crucial factor in harming the function of MSCs^7,8,10,11^. However, the specific associations between ROS and MSC damage have not been fully elucidated.

High ROS levels can injure proteins, lipids and DNA within the mitochondria and impair ATP production, causing energy stress. AMP-activated protein kinase (AMPK), a sensor of cellular energy status, can be activated by metabolic stresses, such as inhibited ATP production or accelerated ATP consumption, to promote mitochondrial concentration and fragmentation^12,13^. Several studies have indicated that abnormal activation of AMPK leads to excessive mitochondrial fragmentation, followed by mitochondrial dysfunction and cell damage^14–17^. Toyama et al.^18^ determined that activated AMPK could induce mitochondrial fission factor (MFF) to facilitate the recruitment of the GTPase dynamin-related protein 1 (Drp1) from the cytosol to the mitochondrial surface and is involved in the process of mitochondrial fission. Drp1 also has been associated with neurotoxicity and the invasion, transformation and migration of tumour cells by promoting aberrant mitochondrial division and fragmentation^19–24^. However, whether and how the AMPK/MFF/Drp1 pathway is associated with the MSC damage caused by IO has rarely been studied in MDS patients.

In the present study, we evaluated apoptosis, viability and mitochondrial morphology of BM-derived MSCs (BM-MSCs) from the HS-27a cell line and MDS patients with IO, and explored the role of the AMPK/MFF/Drp1 pathway in MSC damage in the HS-27a cell line and MDS patients under the condition of IO.

## Results

### IO increased cell apoptosis and inhibited cell viability through upregulation of ROS levels in MSCs

**Fig. 1:**
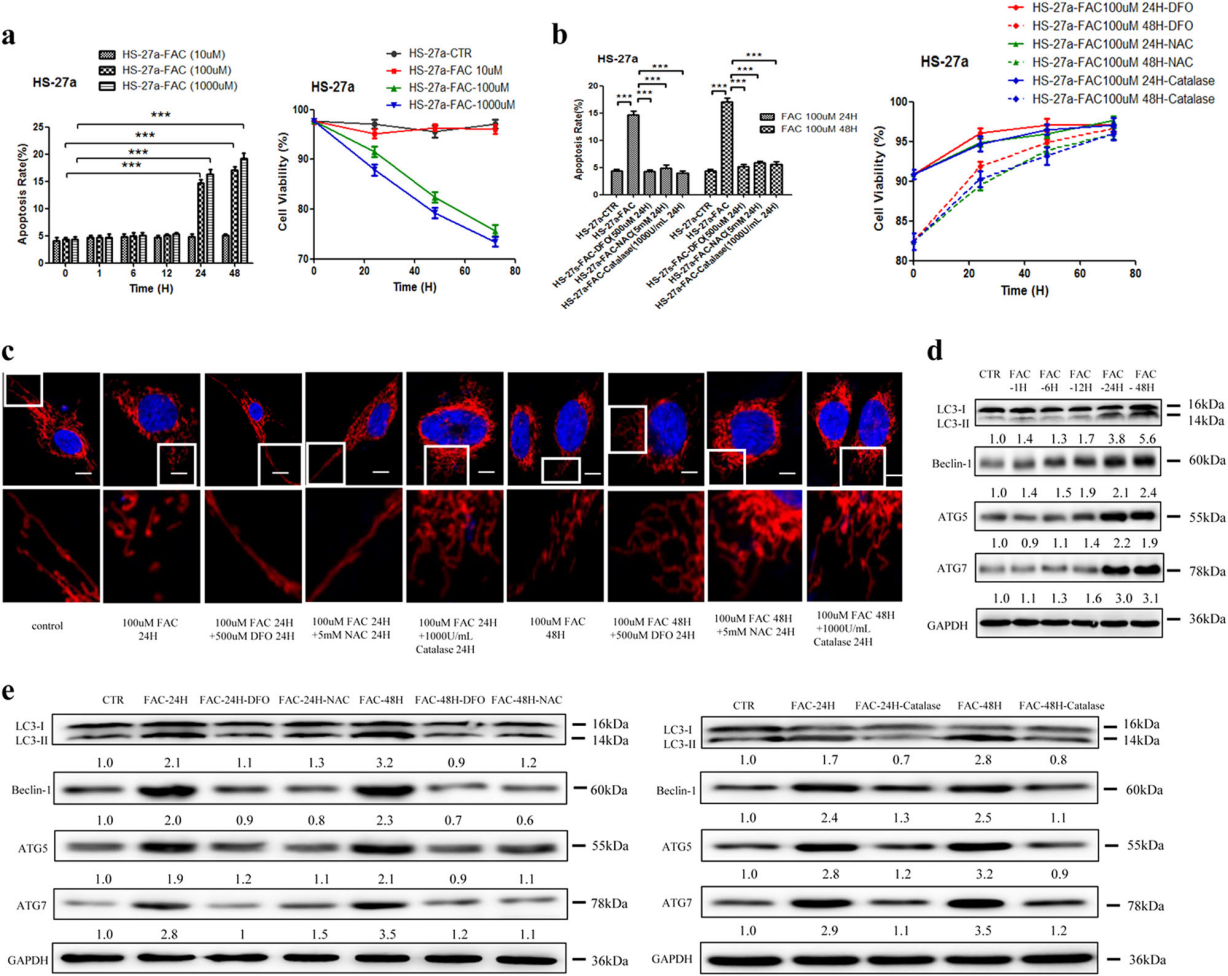
Iron overload impaired the functions of MSCs via up-regulating ROS levels. **a**, **b** The percentage of apoptotic MSCs was tested by Annexin V/PI dual staining and cell viability of MSCs was evaluated by a haemocytometer at 24, 48 and 72 h of culture. **c** Representative confocal images of the mitochondrial morphology in MSCs after incubation with FAC, FAC + DFO, FAC + NAC, FAC +  Catalase or not. Mitochondria were visualized using an antibody to TOM20. Scale bars, 0.5 μm. **d** Western blotting results of LC3, Beclin-1, ATG5 and ATG7 in MSCs before and after adding FAC at 1, 6, 12, 24 and 48h of culture. **e** Western blotting results of LC3, Beclin-1, ATG5 and ATG7 in MSCs before and after adding FAC, FAC + DFO, FAC + NAC or FAC + Catalase. The results were presented as mean ± SD from at least three independent experiments. ***p ≤ 0.001

To confirm the cellular toxicity of iron in MSCs, an IO model was established in the marrow stromal cell line HS-27a with Ferric ammonium citrate (FAC) in vitro. The cellular iron content increased in a time- and concentration-dependent manner after incubation with FAC (Figure S1a). Desferrioxamine (DFO) efficiently decreased the cellular iron content after the addition of 100 µM FAC to HS-27a cells for 24 h under the conditions of 500 µM, 24 h (Figure S1b). High concentrations of iron promoted cell apoptosis and inhibited cell viability, accompanied by increased ROS levels (Fig. 1a and Figure S1c, g). DFO (500 µM, 24 h) successfully decreased the levels of ROS and cell apoptosis caused by IO (Fig. 1b and Figure S1d, g). To define the role of ROS in IO-induced cell apoptosis, *N*-acetyl-l-cysteine (NAC) (an ROS inhibitor) and Catalase (an anti-oxidase) were used (Figure S1e, f and g). Both NAC (5 mM, 24 h) and Catalase (1000 U/ml, 24 h) reversed the effects of IO on HS-27a cells. As shown in Figure 1b, both NAC and Catalase reduced cell apoptosis and enhanced cell viability in IO HS-27a cells. Therefore, these data support the conclusion that IO can injure MSCs by upregulating the ROS levels in MSCs.

### IO caused mitochondrial fragmentation and autophagy enhancement in MSCs

Mitochondria is highly dynamic organelles that have a key role in cellular functions, such as cell apoptosis, cell division and cell cycle^16,25–27^. To study the effects of IO on mitochondrial dynamics in HS-27a cells, we analysed mitochondrial morphological changes. Confocal microscopy images of endogenous TOM20 staining revealed extensive fragmentation of mitochondria induced by FAC (Fig. 1c and Figure S1h). Moreover, LC3-II conversion and the protein levels of Beclin-1, ATG5 and ATG7 were increased in HS-27a cells under an IO environment, as evaluated by western blotting (Fig. 1d). By contrast, the effects of IO on mitochondrial fragmentation and autophagy were significantly attenuated by the addition of DFO, NAC and Catalase to HS-27a cells (Fig. 1c,d,e). These results suggest that IO can cause mitochondrial fragmentation and autophagy enhancement in MSCs, and ROS have an important role in these effects.

### IO reduced ATP levels by inhibiting ETC II/III activity in MSCs

**Fig. 2:**
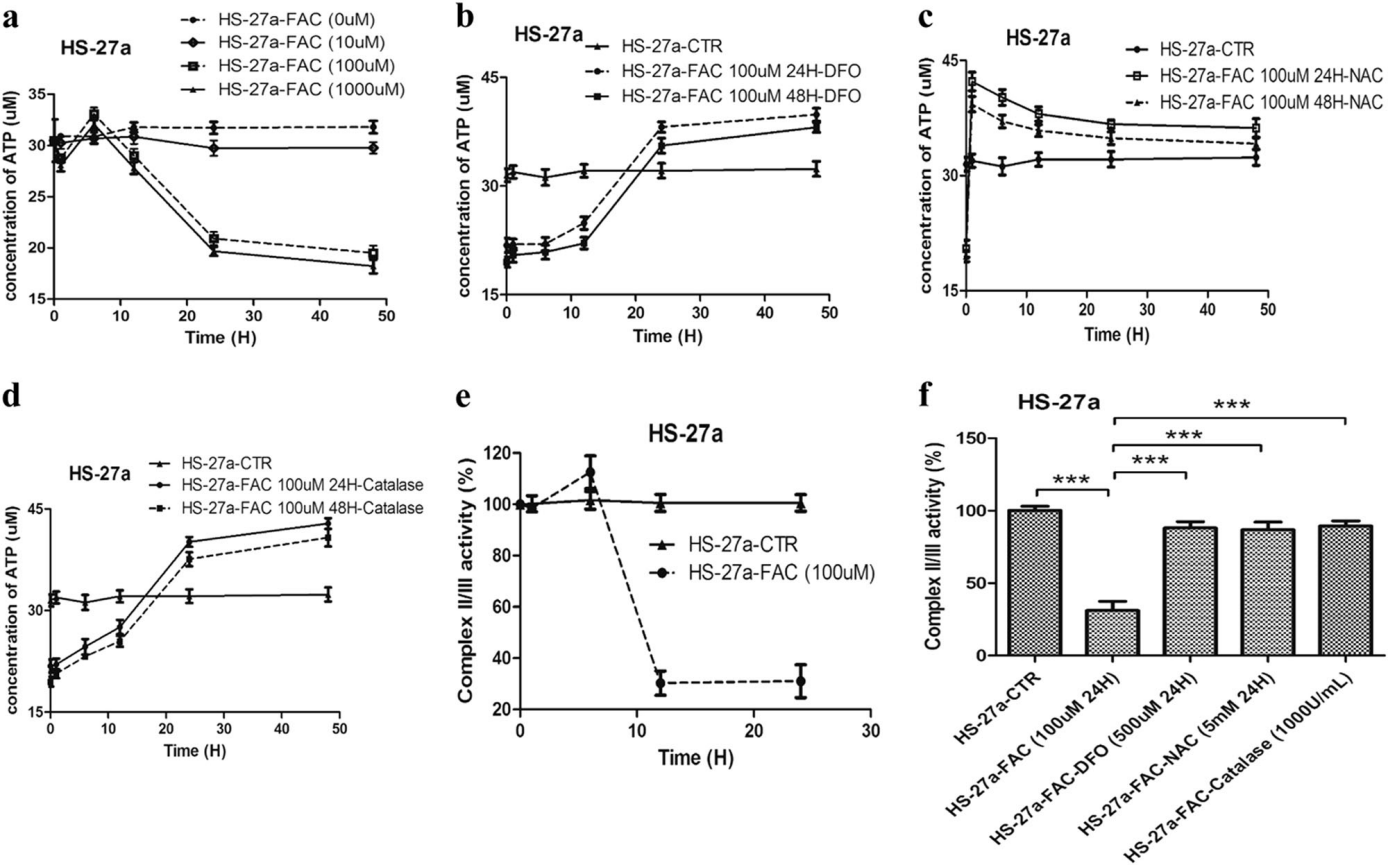
Iron overload caused the reduction of ATP via upregulating ROS levels and inhibiting ETC II/III activity in MSCs. **a** Concentrations of ATP were measured by microplate reader in MSCs after incubation with different concentrations of FAC at 1, 6, 12, 24 and 48 h. **b**–**d** Concentrations of ATP were measured in MSCs with iron overload after adding DFO, NAC or Catalase at 1, 6, 12, 24 and 48 h. **e**, **f** The activity of ETC II/III was detected by microplate reader in MSCs before and after adding FAC, FAC + DFO, FAC + NAC or FAC + Catalase. The results were presented as mean ± SD from at least three independent experiments. ***p ≤ 0.001

Mitochondrial morphological dynamics are closely linked to cellular energy metabolism, and the concentrations of ATP also regulate mitochondrial morphology^28^. To explore whether IO could impact energy metabolism, we investigated ATP levels in HS-27a cells using a microplate reader. We found that the ATP levels were markedly reduced after incubation with high concentrations of iron for 24 and 48 h in HS-27a cells (Fig. 2a). Subsequently, to further analyse the mechanism of ATP reduction caused by IO, we tested the role of ROS and the activity of electron transport chain complex (ETC) I and II/III. We added DFO, NAC and Catalase to IO cells and the addition of DFO, NAC and Catalase obviously increased the ATP levels (Fig. 2b,c,d). In addition, IO inhibited the activity of ETC II/III in HS-27a cells, which could lead to a reduction in ATP levels (Fig. 2e and Figure S2). In addition, DFO, NAC and Catalase substantially improved the activity of ETC II/III (Fig. 2f). In summary, IO can reduce ATP levels via upregulation of ROS levels and inhibition of ETC II/III activity in MSCs.

### IO-induced mitochondrial fragmentation and cellular damage depend on activation of AMPK

**Fig. 3:**
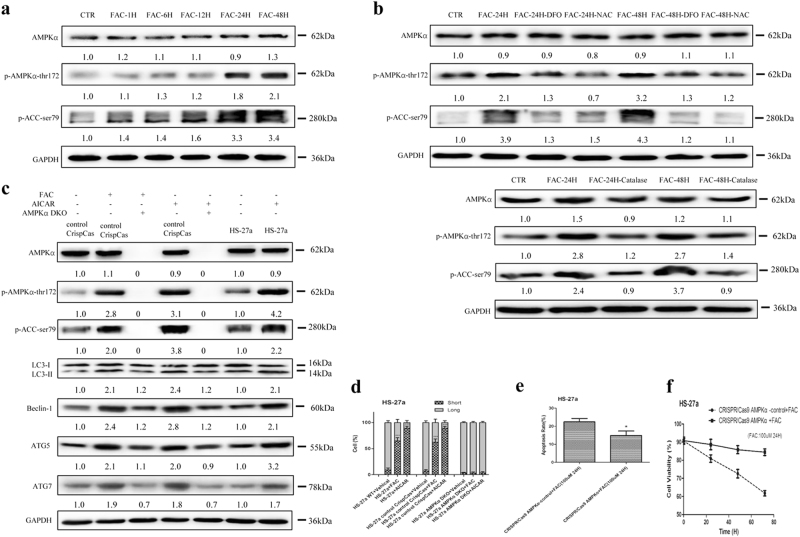
AMPK is involved in iron overload-induced mitochondrial fragmentation in MSCs. **a**, **b** Protein expression of AMPKα, p-AMPKα-thr172 and p-ACC-ser79 were analysed by western blotting after treatment with FAC, FAC + DFO, FAC + NAC or FAC + Catalase in MSCs. **c** Protein expression levels of AMPKα, p-AMPKα-thr172, p-ACC-ser79, LC3, Beclin-1, ATG5 and ATG7 in WT MSCs or control CrispCas MSCs, or AMPKα DKO MSCs before and after treatment with FAC or AICAR. **d** Quantification of the mitochondrial morphology of cells shown in (Supplementary Figure S3c). A total of 100 cells in each experimental group were observed. **e** The percentage of apoptotic MSCs was tested by Annexin V/PI dual staining. **f** Cell viability of MSCs was evaluated by a haemocytometer at 24, 48 and 72 h of culture. The results were presented as mean ± SD from at least three independent experiments. * *p* ≤ 0.05, ***p ≤ 0.001

As a central metabolic sensor, AMPK is activated when intracellular ATP concentrations decrease^29^. Upon activation, AMPK regulates the intracellular energy levels and mitochondrial morphology^30^. However, the question of whether AMPK has a role in IO-induced mitochondrial morphological changes and cellular damage in MSCs has rarely been studied. Thus, we examined the activation of AMPK under IO conditions in HS-27a cells. The results showed that phosphorylation of AMPK (Thr172) and the downstream substrate acetyl coenzyme A carboxylase (ACC) (Ser79) were significantly enhanced by IO (Fig. 3a). In accordance with the ATP results, the addition of DFO, NAC and Catalase decreased the phosphorylation of AMPK (Thr172) and ACC (Ser79) in HS-27a cells with IO (Fig. 3b).

To further confirm that AMPK has an important role in IO-induced cellular function changes, we constructed a CRISPR/Cas9 system to genetically knock out the double catalytic subunit of AMPK (AMPKα1 and AMPKα2) in HS-27a cells (AMPK double-knockout (DKO)). AMPK catalytic subunits were genetically deleted in HS-27a cells to fully abolish AMPK signalling, as evaluated by real-time PCR (Figure S3a) and western blotting (Figure S3b and Fig. 3c). 5-Amino-1-β-d-ribofuranosyl-imidazole-4-carboxamide (AICAR) (2 mM, 1 h), an AMP-mimetic compound, triggered activation of AMPK kinase complexes and mitochondrial fragmentation but not in AMPK DKO HS-27a cells (Fig. 3c,d and Figure S3c)^31^. As predicted, IO-induced mitochondrial fragmentation and cellular functional damage were significantly attenuated in AMPK DKO HS-27a cells (Fig. 3c,d,e,f and Figure S3c). Therefore, activation of AMPK participates in the changes in cellular function induced by IO in MSCs.

### The MFF/Drp1 pathway is activated in MSCs with IO

**Fig. 4:**
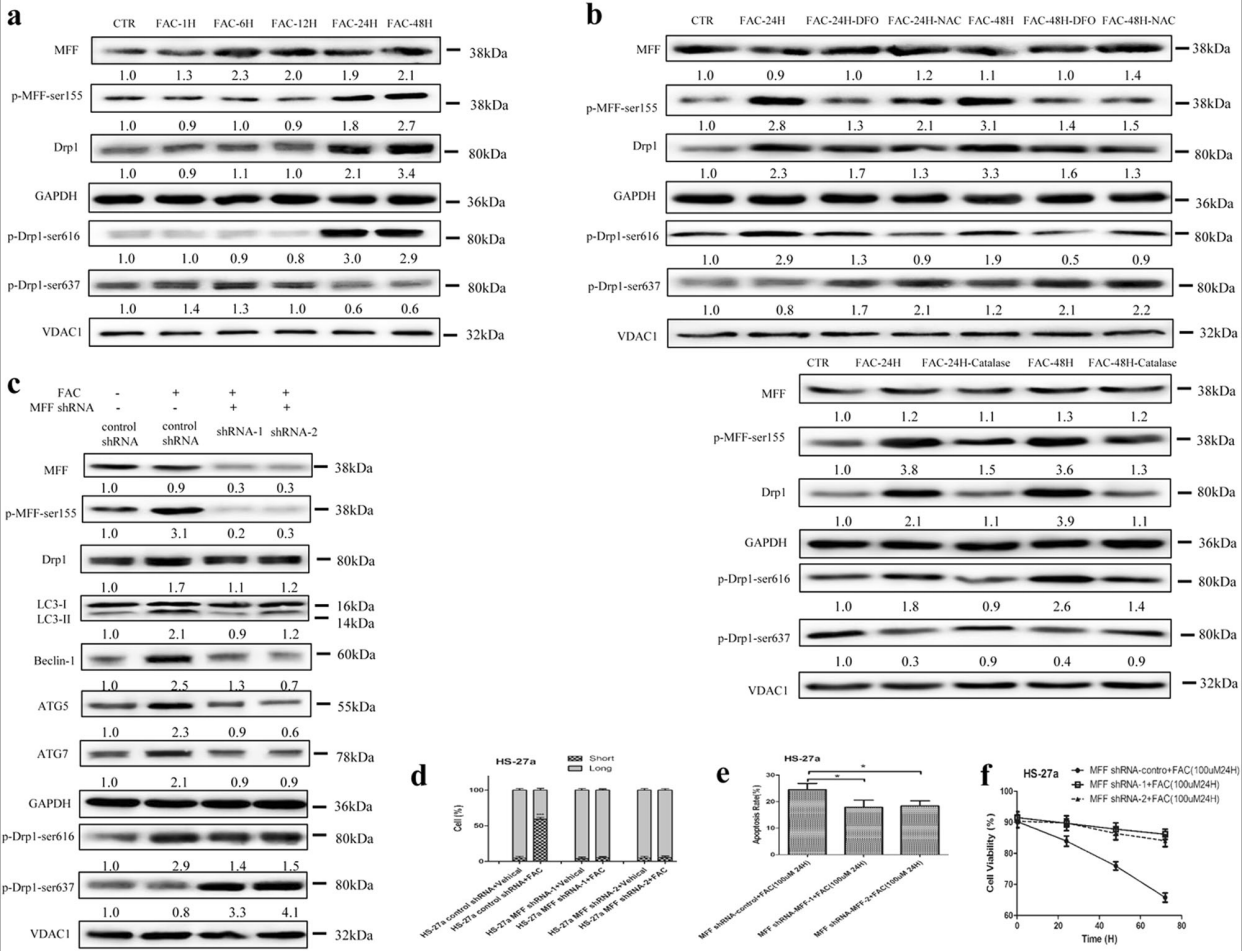
MFF/Drp1 pathway has an important role in iron overload-induced mitochondrial fragmentation in MSCs. **a**, **b** Total protein levels of MFF, p-MFF-ser155, Drp1 and mitochondrial protein levels of p-Drp1-ser616, p-Drp1-ser637 were analysed by western blotting after treatment with FAC, FAC + DFO, FAC + NAC or FAC + Catalase in MSCs. **c** Total protein expression of MFF, p-MFF-ser155, Drp1, LC3, Beclin-1, ATG5, ATG7 and mitochondrial protein expression of p-Drp1-ser616, p-Drp1-ser637 in control shRNA MSCs or MFF shRNA MSCs before and after adding FAC. **d** Quantification of the mitochondrial morphology of cells shown in (Supplementary Figure S4c). A total of 100 cells in each experimental group were observed. **e** The apoptotic rate of MSCs was tested by Annexin V/PI dual staining. **f** Cell viability of MSCs was evaluated by a haemocytometer at 24, 48 and 72 h of culture. The results were presented as mean ± SD from at least three independent experiments. *p ≤ 0.05, ***p ≤ 0.001

Mitochondrial fission involves the recruitment of Drp1 from the cytosol to the mitochondrial surface, to catalyse the fission reaction. As a dominant receptor for Drp1, MFF is a key factor in regulation of mitochondrial morphology^32–34^. In this study, we found that IO increased the phosphorylation of MFF (Ser155) and the total protein levels of Drp1, accompanied by an increase in Drp1-Ser616 phosphorylation and a decrease in Drp1-sSer637 phosphorylation in mitochondria (Fig. 4a). By contrast, DFO, NAC and Catalase attenuated the above results caused by IO (Fig. 4b). Therefore, IO can activate the MFF/Drp1 pathway in MSCs.

To further ascertain that the MFF/Drp1 pathway is involved in IO-induced cellular functional changes, we constructed two retrovirus-based RNA interference vectors to knock down the expression of MFF that transfected HS-27a cells with high efficiency. On average, MFF was reduced by ~ 90%, as evaluated with real-time PCR (Figure S4a) and western blotting (Figure S4b). We found that the total protein levels of Drp1 were downregulated, Drp1-Ser616 phosphorylation was decreased and Drp1-Ser637 phosphorylation was increased in mitochondria after knockdown of MFF in HS-27a cells (Fig. 4c). Importantly, IO-induced mitochondrial fragmentation was completely attenuated, cell apoptosis reduced and cell viability enhanced in MFF knockdown HS-27a cells, accompanied by weakened autophagy (Fig. 4c,d,e,f and Figure S4c). Together, these data indicate that the MFF/Drp1 pathway has a key role in IO-induced cellular functional changes.

### AMPK directly regulates the activity of the MFF/Drp1 pathway in MSCs with IO

**Fig. 5:**
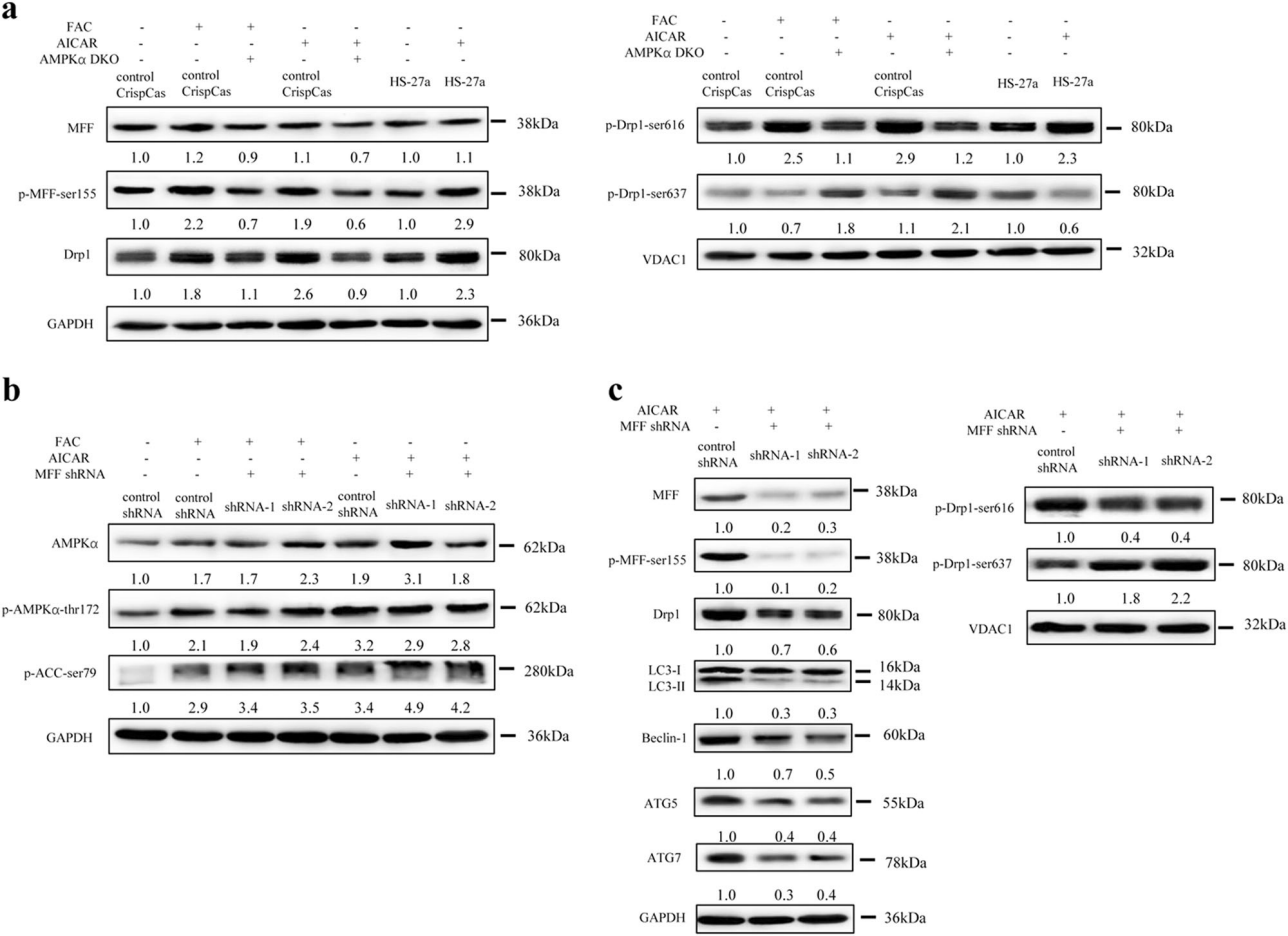
AMPK could directly control MFF/Drp1 pathway in MSCs with iron overload. **a** Total protein expression of MFF, p-MFF-ser155, Drp1 and mitochondrial protein expression of p-Drp1-ser616, p-Drp1-ser637 in WT MSCs or control CrispCas MSCs or AMPKα DKO MSCs before and after treatment with FAC or AICAR. **b** Protein expression of AMPKα, p-AMPKα-thr172 and p-ACC-ser79 in control shRNA MSCs or MFF shRNA MSCs before and after adding FAC or AICAR. **c** Total protein expression of MFF, p-MFF-ser155, Drp1, LC3, Beclin-1, ATG5 and ATG7, and mitochondrial protein expression of p-Drp1-ser616, p-Drp1-ser637 in control shRNA MSCs or MFF shRNA MSCs before and after adding AICAR. The average of three replicates is displayed

Previously, MFF has been established as a downstream substrate of AMPK and can be phosphorylated by AMPK at Ser146, Ser155 and Ser172, as well as at other sites^35–39^. In this study, we further determined whether MFF is an AMPK-dependent substrate in MSCs under IO conditions. Our results revealed that both FAC and AICAR caused robust phosphorylation of MFF and Drp1 in HS-27a cells but not in AMPK DKO HS-27a cells (Fig. 4a and Fig. 5a). However, FAC and AICAR could phosphorylate AMPK and the downstream substrate ACC in MFF knockdown HS-27a cells, even though this was not the cause of MFF/Drp1 pathway activation and mitochondrial fragmentation (Figs. 4c, 5b and 5c, and Figure S4c, d, e). Thus, our data confirm that the MFF/Drp1 pathway is directly controlled by AMPK in MSCs with IO.

### Activation of the AMPK/MFF/Drp1 pathway participates in the damage of MDS-derived MSCs with IO

**Fig. 6:**
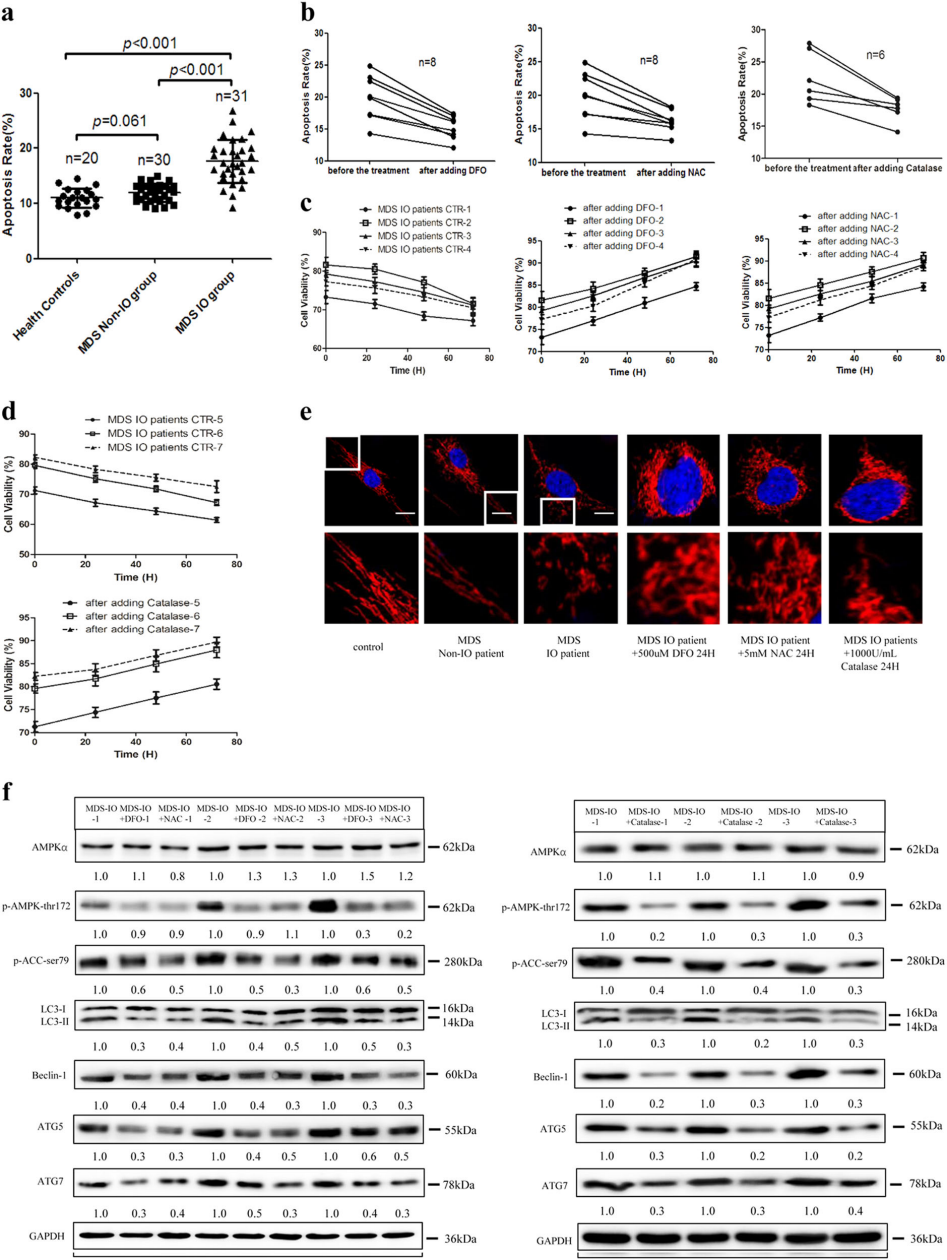
AMPK/MFF/Drp1 pathway participates in the damage of MDS-MSCs with iron overload. **a** The apoptotic rates of MSCs from health controls and MDS patients with or without iron overload. **b** The percentage of apoptotic MDS-MSCs with iron overload before and after adding DFO, NAC or Catalase. **c** Cell viability of MDS-MSCs with iron overload before and after adding DFO, NAC. **d** Cell viability of MDS-MSCs with iron overload before and after adding Catalase. **e** Representative confocal images of the mitochondrial morphology of MSCs from health controls and MDS patients. Mitochondria were visualized using an antibody to TOM20. Scale bars, 0.5 μm. **f** Protein expression of AMPKα, p-AMPKα-thr172, p-ACC-ser79, LC3, Beclin-1, ATG5 and ATG7 in MDS-MSCs with iron overload before and after treatment with DFO, NAC or Catalase. The results were presented as mean ± SD from at least three independent experiments

BM-MSCs cultured from healthy controls (*n* = 28) and MDS patients with (serum ferritin (SF) ≥ 1000 ng/ml, *n* = 41) or without (SF < 1000 ng/ml, *n* = 40) IO were successfully expanded. Then, we performed flow cytometry (FCM) and ATP assays to evaluate the cellular functional features of partial BM-MSCs and found that IO induced higher levels of cell apoptosis in MDS-MSCs, accompanied by upregulation of ROS levels and downregulation of ATP concentrations (Fig. 6a and Figure S5a, b, c, d, e). Furthermore, we compared the above data in “lower-risk” (LR) and “higher-risk” (HR) MDS patients but found no statistically significant differences in cell apoptosis, ROS levels and ATP concentrations in MSCs between LR MDS patients and HR MDS patients (Figure S5f, g and h). However, IO increased cell apoptosis and ROS levels, and decreased ATP concentrations in MSCs from LR MDS patients or HR MDS patients (Figure S5f, g and h). Therefore, our data indicated that IO has an important role in the damage of MSCs from LR or HR MDS patients.

Subsequently, MDS-MSCs from several patients with IO in clinical remission were considered. To explore whether the cell apoptosis- and ATP reduction-inducing effects of IO were mediated via ROS, DFO, NAC and Catalase were introduced to MDS-MSCs with IO. We found that ATP concentrations increased and cell apoptosis decreased after the addition of DFO, NAC and Catalase, and cell viability was enhanced (Fig. 6b,c,d and Figure S5i, j). Thus, our data demonstrate that the effects of IO on MDS-MSCs were ROS dependent.

In addition, confocal microscopy revealed that extensive mitochondrial fragmentation and autophagic enhancement occurred in MDS-MSCs with IO compared with normal MSCs or MDS-MSCs without IO, and the addition of DFO, NAC and Catalase substantially improved those effects (Fig. 6e,f and Figure S5k). Furthermore, we found that IO strongly activated AMPK compared with the addition of DFO, NAC or Catalase in MDS-MSCs, accompanied by an increase in LC3-II conversion and the protein level of Beclin-1, ATG5, ATG7 in three patients out of eight patients (Fig. 6f). These results suggest that IO confers cytotoxicity by activating the AMPK/MFF/Drp1 pathway in MDS-MSCs, which is related to high ROS levels.

## Discussion

In this study, we demonstrated that IO causes extensive mitochondrial fragmentation and autophagy enhancement in MDS-MSCs, which results in MDS-MSCs that are more prone to apoptosis and have a lower viability than BM-MSCs from healthy controls and MDS patients without IO. In MSCs with IO, the ATP concentration was significantly reduced due to the high ROS levels and low ETC II/III activity. Then, the decrease in ATP levels leads to robust AMPK activation that in turn phosphorylates MFF, which recruits the pro-fission molecule Drp1 to mitochondria and allows unopposed mitochondrial fission. Mitochondrial dysfunction caused by the activity of the AMPK/MFF/Drp1 pathway has an important role in cytotoxicity of MDS-MSCs with IO.

Iron toxicity is highly implicated in the progression of MDS and the exact mechanisms are not fully clear. Recently, an increasing number of studies examining the damage induced by IO have focused on the BM microenvironment^6–8,10^. Our study revealed that IO can damage MSCs, including by increasing cell apoptosis and lowering cell viability, both of which are ROS dependent. These results are mostly consistent with previous studies^6–8,10^. More interestingly, MSCs with IO experienced substantial fission of mitochondria and autophagy enhancement, and decreased ROS levels by iron chelation and antioxidants significantly attenuated the effects caused by IO. As previously reported, accumulation of iron in neurons has been proposed to contribute to the pathology of numerous neurodegenerative diseases by destroying mitochondrial morphology^40^. Thus, dysfunction of mitochondrial morphology is of great significance for research into the pathological effects of IO.

The mitochondrial fission and fusion rates respond to changes in energy metabolism^41^. Iron, an essential cofactor for a wide range of cellular processes, is involved in regulation of energy metabolism and oxidative phosphorylation in mitochondria^42^. On the other hand, excess iron can also damage mitochondrial function through a variety of factors, including the formation of high ROS levels and inhibition of some enzymatic activities^11^. In this study, we found that IO induced a markedly reduction in ATP concentration, accompanied by high ROS levels and low ETC II/III activity in MSCs. Inhibition of ETC II/III activity not only impacted the generation of ATP but also may worsen the situation by producing even more ROS^43,44^. High ROS levels can lead to defects in mitochondrial structure by damaging the mitochondrial respiration chain and synthesis of DNA and proteins^11^. This is a vicious circle. However, iron toxicity is highly implicated in the pathological process of ATP reduction and ROS generation, and more studies are needed in the future.

A balance between ATP consumption and ATP generation is one of the fundamental requirements for all cells. AMPK, a highly conserved sensor of intracellular adenosine nucleotide levels, is activated when even modest decreases in ATP production occur. In response, activated AMPK triggers metabolic changes that increase catabolic pathways to generate more ATP and inhibit anabolic pathways to reduce energy consumption^30,45^. However, in response to severe stress, activated AMPK can directly promote mitochondrial fission and cell autophagy to govern mitochondrial morphology and cellular function^30,41^. Whether AMPK is involved in IO-induced mitochondrial fragmentation in MSCs has been rarely studied. In our research, we found that IO phosphorylated AMPK and its downstream substrate ACC, and DFO, NAC and Catalase reversed the effects caused by IO. More importantly, neither FAC nor AICAR could induce phosphorylation of AMPK and ACC or induce mitochondrial fragmentation or autophagy enhancement in AMPK DKO MSCs. Therefore, we demonstrated that AMPK is closely related to IO-induced mitochondrial fragmentation and autophagy enhancement.

Drp1 is the main factor that mediates the process of mitochondrial fission through phosphorylation of Ser616 and dephosphorylation of Ser637^21,40^. In mammals, Drp1 is recruited from the cytosol to mitochondrial fission sites by Mid49, Mid51, FIS1 and MFF^46^. However, abnormal activation of Drp1 can cause destruction of mitochondria, and migration and invasion of some tumours^22,23^. AMPK can regulate Drp1 phosphorylation to impact mitochondrial fragmentation in pancreatic β cells^47^. Furthermore, MFF has been reported by several studies to be an AMPK-dependent substrate^18,35^. Thus, we hypothesised that AMPK is involved in IO-induced mitochondrial fragmentation and autophagy enhancement by directly controlling the MFF/Drp1 pathway in MSCs. Our results found that the MFF/Drp1 pathway is activated by IO and has a key role in IO-induced mitochondrial fragmentation and autophagy enhancement in MSCs. Importantly, the activation of the MFF/Drp1 pathway induced by FAC and AICAR was cancelled in AMPK DKO MSCs, and the same results were observed for mitochondrial fragmentation and autophagy enhancement. In addition, AMPK and ACC can be phosphorylated by FAC and AICAR in MFF knockdown MSCs, although they do not cause mitochondrial fragmentation. Therefore, our data suggest that the activation of the AMPK/MFF/Drp1 pathway has a critical role in promoting the mitochondrial fragmentation and autophagy caused by IO in MSCs.

Subsequently, we further validated the mechanism of the damage caused by IO in MDS-MSCs. The results revealed that IO caused higher cell apoptosis and lower cell viability in MDS-MSCs, which were related to high ROS levels and low ATP concentrations. Moreover, the above IO-induced effects of MDS-MSCs were not related to LR or HR. In addition, DFO, NAC or Catalase weakened the activation of AMPK caused by IO in MDS-MSCs from several patients, accompanied by attenuation of mitochondrial fragmentation and autophagy. Therefore, our results demonstrate that the AMPK/MFF/Drp1 pathway is involved in the cellular functional damage of MDS-MSCs induced by IO.

In summary, we investigated the molecular mechanisms of the damage caused by IO in MDS-MSCs. Our data demonstrate that IO promotes mitochondrial fragmentation and cell apoptosis, and inhibits cell viability in MDS-MSCs through high ROS levels and low ETC II/III activity that in turn reduce ATP concentrations. Then, reduced ATP concentrations further activate the AMPK/MFF/Drp1 pathway and activation of AMPK/MFF/Drp1 pathway has an important role in the damage of MDS-MSCs caused by IO. Our study provides a basis and a new direction for further research and treatment of MDS patients.

## Materials and methods

### Patients

In total, 81 MDS patients were enrolled in this study. All patients were untreated when they were recruited into this study. The patients were diagnosed with MDS according to the minimum diagnostic criteria established by the conference on MDS^48^ and classified as LR (International Prognostic Scoring System) (IPSS)-low/int-1) or HR (IPSS-int-2/high)^49^. Their characteristics are detailed in Table S1. In total, 28 healthy volunteers were used as controls and were matched for gender and age. All study participants provided informed consent form in accordance with the Declaration of Helsinki and the research was approved by the ethics committee of the Sixth Hospital affiliated with Shanghai Jiao Tong University.

### Cell line and culture

The human BM stromal cell line HS-27a was cultured in RPMI-1640 medium with 10% fetal bovine serum and penicillin (100 units/ml)/streptomycin (100 µg/ml) at 37 °C in a humidified atmosphere with 5% CO_2_.

### Isolation and culture of BM-MSCs

BM mononuclear cells were separated with a Ficoll-Paque Plus (GE Healthcare, Uppsala, Sweden) and cultured in Human Mesenchymal Stem Cell Growth Medium (Cyagen Biosciences, Inc., Guangzhou, China) with 10% fetal bovine serum, glutamine and 100 U/ml penicillin–streptomycin at 37 °C with 5% CO_2_ in a fully humidified atmosphere. The supernatant containing non-adherent cells was removed and replaced with fresh supplemented medium every 3–4 days. Upon achieving > 80–90% confluence, the cells were detached with 0.25% trypsin-EDTA (Gibco, Grand Island NY, USA). At the third passage (P3), MSCs were collected and utilized for experimental research.

### Reagents

FAC, calcein acetoxymethyl ester (CA-AM), deferiprone (L1) and 2',7'-dichlorofluorescin diacetate (DCFH-DA) were purchased from Sigma-Aldrich (USA). DFO was from Novartis Pharma (Switzerland). NAC, Catalase, Beclin-1, ATG5 and ATG7 were from Beyotime Biotechnology (Shanghai, China). AICAR was from Med Chem Express (USA). AMPK, p-AMPK (Thr172), p-Drp1 (Ser616), p-Drp1 (Ser637), p-ACC (ser79), LC3, GAPDH and VDAC1 were from Cell Signalling Biotechnology (Danvers, MA, USA). MFF was from Abcam (Cambridge, MA, USA). p-MFF (ser155) was generated by YenZym Antibodies (South San Francisco, CA, USA). TOM20 and Drp1 were from Santa Cruz Biotechnology, Inc. (Santa Cruz, CA, USA). Alexa Fluor 594-conjugated goat anti-rabbit IgG (H + L) secondary antibody, Alexa Fluor 488-conjugated goat anti-mouse IgG (H + L) secondary antibody and MitSOX^TM^ Red Mitochondrial Superoxide Indicator were from Thermo Scientific (Rockford, IL, USA).

### Labile iron pool assay

Cells were incubated with 5 μM CA-AM for 30 min at 37 °C in the dark, washed with phosphate-buffered saline (PBS) and treated with 1 mM L1 for 1 h or left untreated at 37 °C. The stained cells were analysed with a FCM (Becton Dickinson, USA). The labile iron pool was reflected by the difference in cellular fluorescence before and after incubation with L1 (Δ*F* = *F*_after_ − *F*_before_)^50^.

### ROS assay

For cellular total ROS levels: cells were collected and were incubated with 10 µM DCFH-DA for 30 min at 37 °C in the dark and washed with PBS. Then, the mixtures were examined via FCM. For mitochondrial ROS levels: cells, seeded in 6-hole plate, were incubated with 1.0–2.0 ml of 5 μM solution of MitoSOXred for 10 min at 37 °C in the dark and washed with PBS. Then, stained with DAPI (4',6-diamidino-2-phenylindole) and analysed using a confocal microscope (Carl Zeiss AG, Jena, Germany).

### Apoptosis assay

The proportion of apoptotic cells was analysed with Annexin V/propidium iodide dual staining (Multi Sciences Biotech, Co., Ltd, Hangzhou, China) according to the manufacturer’s instructions. The stained cells were examined via FCM.

### Viability assay

A mixture of one part 0.4% trypan blue and one-part cell suspension (1:9) was prepared. A drop of the mixture was applied to a haemocytometer. The average number of total cells and unstained (viable) cells was determined. The rate of cell viability was determined as follows: (average viable cell count/average total cell count) × 100%.

### ATP assay

The ATP levels of cells were determined using an ATP determination kit (Beyotime Biotechnology) according to the manufacturer’s instructions. Luminescence was analysed with a Multiskan Mk3 Microplate Reader (Thermo Scientific).

### Mitochondrial respiratory chain complexes assay

The activities of the ETCs I and II/III were detected with MitoCheck ®complex I or II/III activity assay kits (Cayman Chemical, USA) according to the manufacturer’s instructions. Luminescence was read with a Multiskan Mk3 Microplate Reader.

### Mitochondrial morphology assay

Cells were seeded onto 20 mm round coverslips and fixed with 4% paraformaldehyde for 20 min. Then, each well was washed with PBS and blocked with 1% bovine serum albumin for 1 h. Primary antibodies were incubated overnight at 4 °C and cells were exposed to the corresponding secondary antibodies for 1 h at 37 °C, then washed with PBS and stained with DAPI. Images were analysed with an LSM 710 confocal microscope (Carl Zeiss AG) and mitochondrial length was measured with ImageJ software (long: > 3 µm, short: ≤ 3 µm) (NIH, Bethesda, MD, USA)^51^.

### Real-time PCR assay

Total RNA was extracted using an RNeasy Mini Kit (Qiagen, Hilden, Germany) and a Revert Aid TM First Strand cDNA Synthesis Kit (Fermentas, Burlington, Canada) was used to synthesise cDNA by following the relevant manufacturer’s protocol. PCR was performed using Real Master Mix (Takara, Dalian, China) on an ABI7500 real-time PCR machine (Applied Biosystems, Foster, CA, USA). The primer sequences are listed in Table S2.

### Western blotting assay

Equal quantities of total or mitochondrial protein were separated via SDS-polyacrylamide gel electrophoresis and transferred to polyvinylidene fluoride membranes. Primary antibodies were incubated with the membranes overnight at 4 °C, followed by exposure to the corresponding secondary antibodies for 1 h. The proteins were visualized with enhanced chemiluminescence (Bio-Rad, USA). The films were scanned using a Epson Perfection 4490 Scanner (EPSON Europe B.V., Sweden).

### Virus transfection assay

CRISPR/Cas9-AMPKα (AMPKα1 and AMPKα1) and shRNA-MFF viruses were prepared by Genechem Company (Shanghai, China), and the transduction was performed according to the manufacturer’s protocol.

### Statistical analysis

All statistical analyses were performed using SPSS 17.0 software. The results are presented as the mean ± SD. Student’s *t*-test or one-way analysis of variance was used to analyse the data. A *p*-value < 0.05 was considered to be statistically significant and is indicated on graphs by **p-value < 0.01 and **p-value < 0.001, respectively.

## Electronic supplementary material

The main characteristics of patients with MDS who were enrolled in the study

Primer sets for RT-PCR

Iron content and ROS levels were detected

The activity of electron transport chain complex I

The expression levels of AMPK and mitochondrial morphology

The expression levels of MFF and mitochondrial morphology

Reduced ATP concentrations were related with high ROS levels in MDS-MSCs with iron overload

Reduced ATP concentrations were related with high ROS levels in MDS-MSCs with iron overload

Supplementary figure legends
