# Supplementary material for: Iron overload promotes mitochondrial fragmentation in mesenchymal stromal cells from myelodysplastic syndrome patients through activation of the AMPK/MFF/Drp1 pathway
Source: Cell Death Dis. 2018 May 3;9(5):515. doi: 10.1038/s41419-018-0552-7 (PMC5938711; doi:10.1038/s41419-018-0552-7)
Supplement: Supplementary file 1 — The main characteristics of patients with MDS who were enrolled in the study [file 41419_2018_552_MOESM1_ESM.doc]

**Supplementary table S1** The main characteristics of patients with MDS who were enrolled in the study

| Parameter IO group (n=41) Non-IO group (n=40) *P* value |
| --- |
| Median age, years (range) 63 (24-87) 64(38-83) 0.684  Sex, male/female 26/15 25/15 0.932  Hemoglobin, g/L, median (range) 70 (34-130) 79 (50-144) 0.038  WBC, × 10^9^/L, median (range) 2.8 (1-19.9) 3.5 (0.9-19.3) 0.421  NC, %, median (range) 42.7 (6-86.2) 55.5 (25.5-87.3) 0.065  Platelet, ×10^9^/L, median (range) 36(1-209) 59(1-690) 0.001  Diagnosis by WHO 2008 classification (%)  RCUD 12 13  RCMD 46 52  RARS 8 5  RAEB-1/RAEB-2 34 30  IPSS score (%) 0.771  Low/Int-1 risk 61 63  Int-2/high risk 39 37 |

*Abbreviations*: *WBC* white blood count, *NC* neutrophils count, *WHO* World Health Organization, *RCUD* refractory cytopenia with unilineage dysplasia, *RCMD* refractory cytopenia with multilineage dysplasia, *RARS* refractory anemia with ringed sideroblasts, *RAEB* refractory anemia with excess of blasts, *IPSS* International Prognostic Scoring System, *RBC* red blood count, *IO* iron overload
