# Supplementary material for: Iron overload promotes mitochondrial fragmentation in mesenchymal stromal cells from myelodysplastic syndrome patients through activation of the AMPK/MFF/Drp1 pathway
Source: Cell Death Dis. 2018 May 3;9(5):515. doi: 10.1038/s41419-018-0552-7 (PMC5938711; doi:10.1038/s41419-018-0552-7)
Supplement: Supplementary file 2 — Primer sets for RT-PCR [file 41419_2018_552_MOESM2_ESM.doc]

**Supplementary table S2** Primer sets for RT-PCR

| **Name Primer Sequence (5’ to 3’)** |
| --- |
| AMPKα1-F TTGAAACCTGAAAATGTCCTGCT |
| AMPKα1-R GGTGAGCCACAACTTGTTCTT |
| AMPKα2-F GTGAAGATCGGACACTACGTG |
| AMPKα2-R CTGCCACTTTATGGCCTGTTA |
| MFF-F ACTGAAGGCATTAGTCAGCGA |
| MFF-R TCCTGCTACAACAATCCTCTCC |
| GAPDH-F GCACCGTCAAGGCTGAGAAC |
| GAPDH-R GTGGTGAAGACGCCAGTGGA |

*Abbreviations*: *AMPKα1* protein kinase AMP-activated catalytic subunit alpha 1, *AMPKα2* protein kinase AMP-activated catalytic subunit alpha 2, *MFF* mitochondrial fission factor, *GAPDH* glyceraldehyde-3-phosphate dehydrogenase
