# Supplementary figures and images for: Iron overload promotes mitochondrial fragmentation in mesenchymal stromal cells from myelodysplastic syndrome patients through activation of the AMPK/MFF/Drp1 pathway
Source: Cell Death Dis. 2018 May 3;9(5):515. doi: 10.1038/s41419-018-0552-7 (PMC5938711; doi:10.1038/s41419-018-0552-7)

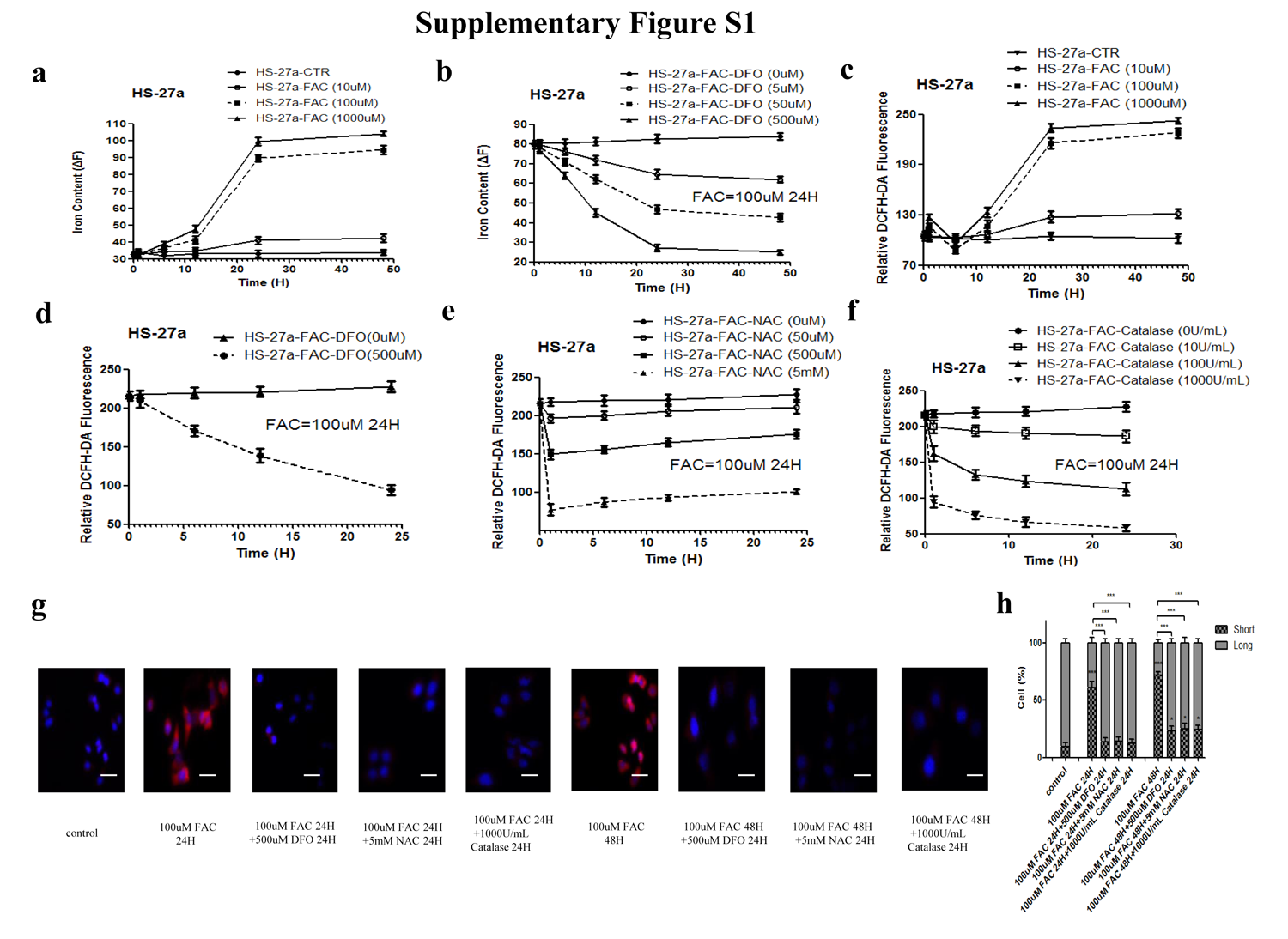

Supplement: Supplementary file 3 — Iron content and ROS levels were detected [file 41419_2018_552_MOESM3_ESM.tif]

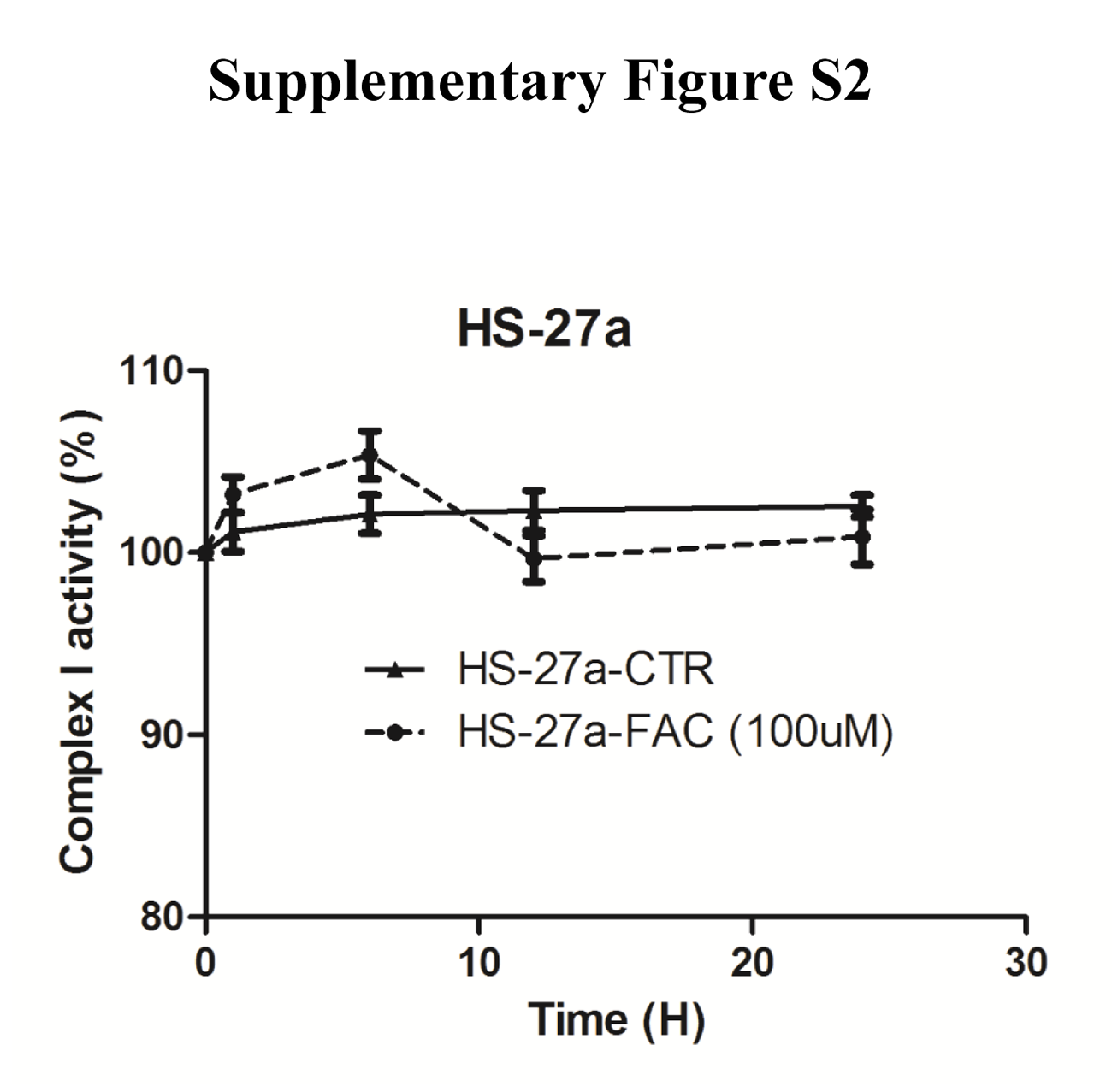

Supplement: Supplementary file 4 — The activity of electron transport chain complex I [file 41419_2018_552_MOESM4_ESM.tif]

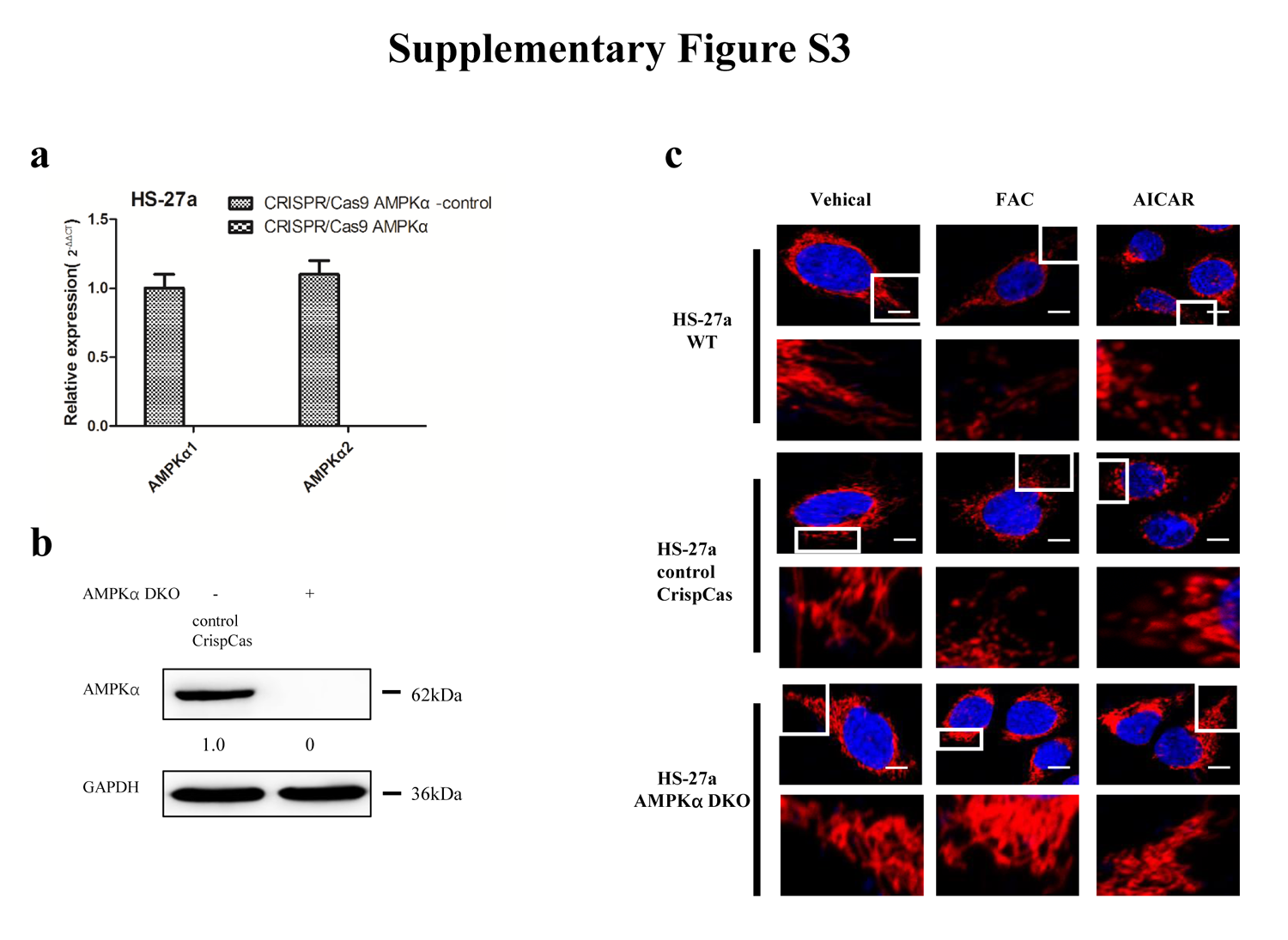

Supplement: Supplementary file 5 — The expression levels of AMPK and mitochondrial morphology [file 41419_2018_552_MOESM5_ESM.tif]

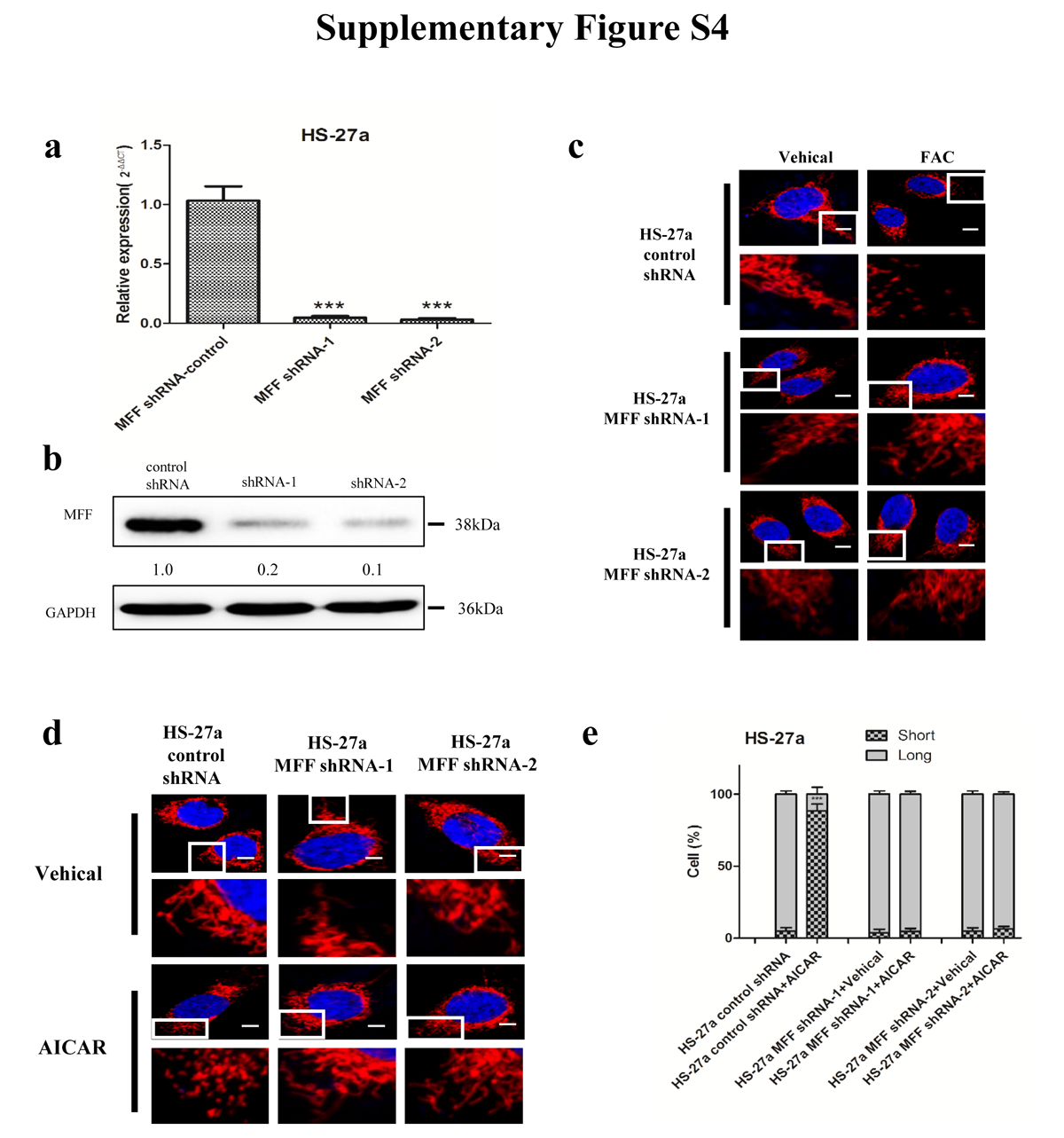

Supplement: Supplementary file 6 — The expression levels of MFF and mitochondrial morphology [file 41419_2018_552_MOESM6_ESM.tif]

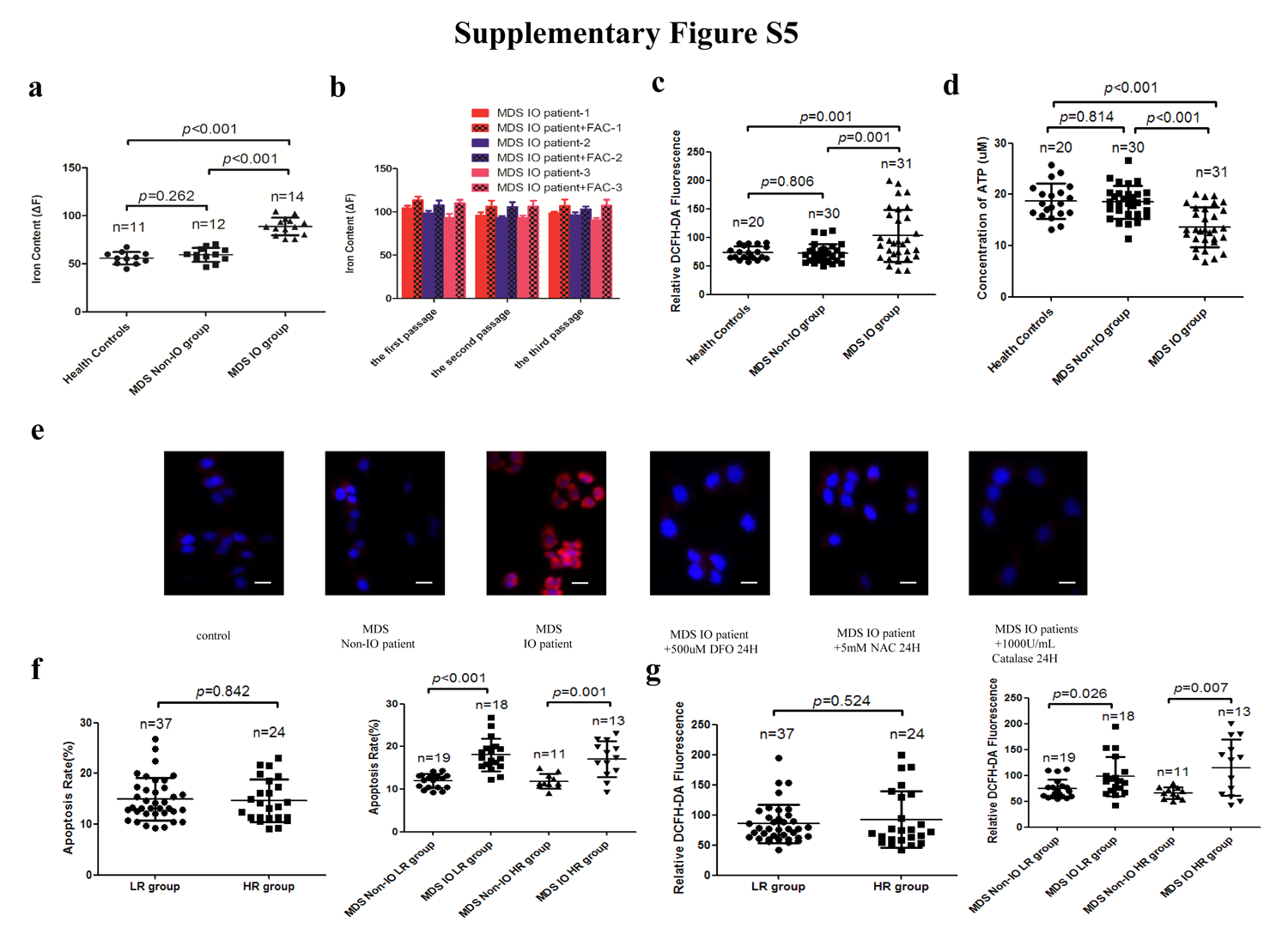

Supplement: Supplementary file 7 — Reduced ATP concentrations were related with high ROS levels in MDS-MSCs with iron overload [file 41419_2018_552_MOESM7_ESM.tif]

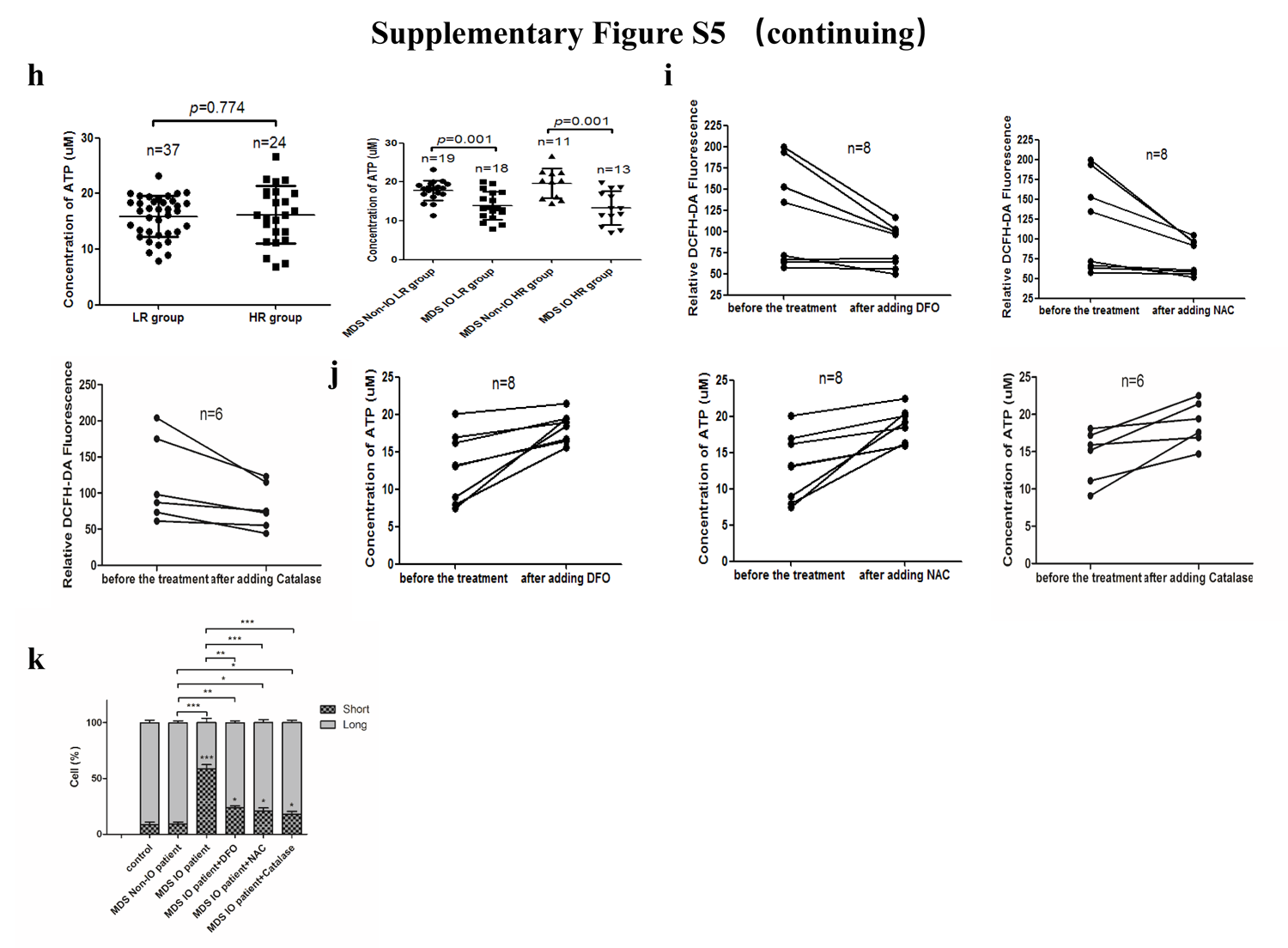

Supplement: Supplementary file 8 — Reduced ATP concentrations were related with high ROS levels in MDS-MSCs with iron overload [file 41419_2018_552_MOESM8_ESM.tif]
