# Supplementary material for: Iron overload promotes mitochondrial fragmentation in mesenchymal stromal cells from myelodysplastic syndrome patients through activation of the AMPK/MFF/Drp1 pathway
Source: Cell Death Dis. 2018 May 3;9(5):515. doi: 10.1038/s41419-018-0552-7 (PMC5938711; doi:10.1038/s41419-018-0552-7)
Supplement: Supplementary file 9 — Supplementary figure legends [file 41419_2018_552_MOESM9_ESM.doc]

**FigureS1 Iron content and ROS levels were detected.** (**a, b**) Cellular iron content of MSCs was detected by CA-AM staining at 1h, 6h, 12h, 24h and 48h. (**c, d, e, f**) ROS levels of MSCs were detected by DCFH-DA staining at 1h, 6h, 12h, 24h and 48h. (**g**) Representative confocal images of the mitochondrial ROS in MSCs after incubation with FAC, FAC+DFO, FAC+NAC, FAC+ Catalase or not. Mitochondrial ROS were visualized using MitoSOX^TM^ reagent. Scale bars, 10um. (**h**) Quantification of the mitochondrial morphology of cells shown in (Figure1c). A total of 100 cells in each experimental group were observed. The results were presented as mean ± SD from at least three independent experiments. *** *p*≤ 0.001.

**FigureS2 The activity of electron transport chain complex I.** The activity of ETC I was detected by microplate reader in MSCs before and after incubation with 100um FAC at 1h, 6h, 12h and 24h. The results were presented as mean ± SD from at least three independent experiments.

**FigureS3 The expression levels of AMPK and mitochondrial morphology.** (**a, b**) The expression levels of AMPKα mRNA (a) and proteins (d) after CRISPR/Cas9 AMPK transfection in MSCs. (**c**) Representative confocal images of the mitochondrial morphology of MSCs after incubation with FAC or AICAR or not in WT MSCs or control CrispCas MSCs or AMPKα DKO MSCs. Mitochondria were visualized using an antibody to TOM20. Scale bars, 0.5um. The results were presented as mean ± SD from at least three independent experiments.

**FigureS4 The expression levels of MFF and mitochondrial morphology.** (**a, b**) The expression levels of MFF mRNA (a) and proteins (b) after MFF shRNA virus transfection in MSCs. (**c**) Representative confocal images of the mitochondrial morphology of MSCs after incubation with FAC or not in control shRNA MSCs or MFF shRNA MSCs. (**d**) Representative confocal images of the mitochondrial morphology of MSCs after incubation with AICAR or not in control shRNA MSCs or MFF shRNA MSCs. (**e**) Quantification of the mitochondrial morphology of cells shown in (d). Mitochondria were visualized using an antibody to TOM20. Scale bars, 0.5um. The results were presented as mean ± SD from at least three independent experiments. *** *p*≤ 0.001.

**FigureS5 Reduced ATP concentrations were related with high ROS levels in MDS-MSCs with iron overload.** (**a**) Cellular iron content of MSCs from health controls and MDS patients with or without iron overload. (**b**) Cellular iron content of MSCs from MDS patients with iron overload before and after incubation with FAC or not. (**c**) ROS levels of MSCs from health controls and MDS patients with or without iron overload. (**d**) ATP concentrations of MSCs from health controls and MDS patients with or without iron overload. (**e**) Representative confocal images of the mitochondrial ROS of MSCs from health controls and MDS patients. Mitochondrial ROS were visualized using MitoSOX^TM^ reagent. Scale bars, 10um. (**f**) The apoptotic rates of MSCs from LR and HR MDS patients. (**g**) ROS levels of MSCs were detected by DCFH-DA staining from LR and HR patients with MDS. (**h**) ATP concentrations of MSCs were detected in LR and HR patients with MDS. (**i**) ROS levels of MDS-MSCs with iron overload were tested before and after adding DFO, NAC or Catalase. (**j**) ATP concentrations of MDS-MSCs with iron overload were tested before and after adding DFO, NAC or Catalase. (**k**) Quantification of the mitochondrial morphology of cells shown in (Figure6e). Mitochondria were visualized using an antibody to TOM20. Scale bars, 0.5um. The results were presented as mean ± SD from at least three independent experiments. **p*≤ 0.05, *** *p*≤ 0.001.
